# Comparison of Single, Fixed-Time Artificial Insemination in Gilts Using Two Different Protocols to Synchronize Ovulation

**DOI:** 10.3390/ani10020306

**Published:** 2020-02-14

**Authors:** Lima Rodrigues, Rocio Amezcua, Glen Cassar, Terri L O’Sullivan, Robert Friendship

**Affiliations:** Department of Population Medicine, University of Guelph, Guelph, ON N1G2W1, Canada; lrodrigu@uoguelph.ca (L.R.); mamezcua@uoguelph.ca (R.A.); tosulliv@uoguelph.ca (T.L.O.); rfriends@ovc.uoguelph.ca (R.F.)

**Keywords:** fixed-time artificial insemination, gilt breeding

## Abstract

**Simple Summary:**

It is necessary to have a consistent supply of service-ready gilts available to incorporate into each batch of breeding sows. Techniques to manipulate the timing of estrus and possibly the timing of ovulation in gilts are helpful in order to achieve this goal. This study investigated two different techniques for inducing ovulation to allow fixed-time artificial insemination (FTAI) protocols in gilts and compared results with gilts bred when observed in standing heat following cessation of daily altrenogest treatment. Pubertal gilts (*n* = 180) were assigned to one of three treatment groups. Group 1 gilts (LUT, *n* = 62) were induced to ovulate utilizing intramuscular injections of equine chorionic gonadotropin followed by porcine luteinizing hormone, and bred using a single FTAI. Group 2 gilts (TRI, *n* = 61) were induced to ovulate by intravaginal deposition of triptorelin acetate and were bred by a single FTAI. Group 3 gilts (CON, *n* = 57) were observed for estrus and bred twice (24 h apart) using artificial insemination (AI). LUT and TRI gilts completed farrowing in a smaller window of time compared to CON gilts; however, they also tended to have poorer reproductive performance. LUT and TRI piglets were 80 g and 64 g, respectively, heavier at weaning than CON piglets. Results indicate that FTAI might be useful as a means of minimizing the range in lactation length in a farrowing batch. However, modifications of the protocols may be required to ensure optimum farrowing rates and litter size.

**Abstract:**

In order to efficiently have a consistent supply of service-ready gilts available to incorporate into each batch of breeding sows, it is necessary to manipulate the timing of estrus and possibly the timing of ovulation of gilts. Estrus can be synchronized by the withdrawal of altrenogest after at least 14 days of treatment. It is possible that protocols developed to induce ovulation, and therefore allow fixed-time artificial insemination (FTAI), can improve the predictability of gilt breeding. This study investigated the effect of two FTAI protocols in gilts on reproductive performance and timing of farrowing and piglet weaning weight compared to gilts bred based on signs of estrus after cessation of altrenogest. Puberty was induced in gilts, followed by treatment with altrenogest. Following altrenogest withdrawal, 180 gilts were assigned to one of three treatment groups. Group 1 gilts (LUT, *n* = 62) were treated with 600 IU equine chorionic gonadotropin 24 h after altrenogest withdrawal and 5 mg porcine luteinizing hormone (pLH) 80 h later, followed by a single FTAI 36 h after pLH. Group 2 gilts (TRI, *n*= 61) received 2 mL of a gonadotropin-releasing hormone agonist, triptorelin acetate, intravaginally 6 d after altrenogest withdrawal and were bred by a single FTAI 24 h later. Group 3 gilts (CON, *n* = 57) were observed for estrus and bred twice by AI, 24 h apart. LUT and TRI gilts farrowed closer together (2.4 ± 1.6 and 2.9 ± 1.2 d(days), respectively) compared to CON gilts (4.5 ± 3.3 d). Piglets in LUT were 80 g (*p* < 0.001) heavier and piglets in TRI were 64 g (*p* < 0.05) heavier at weaning than CON piglets, when controlling for birth weight. Results indicate that FTAI might be useful as a means of minimizing the time from the first to the last gilt farrowing in a breeding batch of gilts. However, modifications of the protocols may be required to ensure optimum farrowing rates and litter size.

## 1. Introduction

In swine production, the performance of the breeding herd determines the flow of pigs throughout the various stages to market. The entry of gilts into the breeding herd is a key factor in maintaining a consistent number of farrowings and meeting breeding targets [1]. Pork producers need to be able to manipulate the estrous cycle of gilts so that there are always an appropriate number of gilts available at each breeding period. This is especially true when a farm employs batch farrowing [2]. Since 30% to 50% of sows are replaced annually [3], efficient control of gilt reproduction can have a great impact on the overall performance of a swine enterprise [1]. Producers may be able to control the timing of estrus and ovulation through synchronization protocols, allowing for the implementation of a single fixed-time artificial insemination (FTAI) program. These programs have been shown to be successful in sows [4,5], but there are few studies that have evaluated FTAI in gilts [4,6].

Puberty can be induced by providing daily boar exposure and this can be enhanced by using a combination of equine chorionic gonadotropin (eCG) and human chorionic gonadotropin (hCG) given as a single intramuscular (IM) injection to pre-pubertal gilts (PG600^®^) [7,8]. Gilts that are cycling can be synchronized by providing a daily oral dose of a progesterone-like product, altrenogest, for at least 14 d. When the altrenogest treatment is withdrawn, gilts will be in estrus within 4 to 9 d [4,9]. In mature sows, an injection of eCG at weaning followed 3 d later with an injection of porcine luteinizing hormone (pLH) can be used to induce ovulation within 35 to 40 h and, therefore, may possibly be used in an FTAI program for gilts [10,11]. Likewise, a gonadotropin-releasing hormone (GnRH) agonist, triptorelin acetate, has also been used to induce ovulation allowing FTAI in sows [5,8], although to date this product does not have a label claim for gilts in Canada. There is a need to investigate these two FTAI methods in gilts to determine whether the use of these techniques might improve estrus and ovulation synchronization in gilts.

The primary objective of the present study was to determine the effect that synchronization protocols have, using either eCG followed by pLH or an intravaginal treatment of triptorelin acetate and a single FTAI, on reducing the time between the first and last farrowings in a batch of gilts and whether this subsequently results in heavier weaning weights of piglets. A secondary objective was to determine if a single FTAI can result in reproductive performance that is comparable to conventional heat detection and double mating without exogenous hormonal induction of ovulation in gilts.

## 2. Materials and Methods

This research trial was approved by the Animal Care Committee of the University of Guelph, in accordance with the guidelines set forward by the Canadian Council of Animal Care.

### 2.1. Pigs and Treatment Groups

This research trial was conducted at the Arkell Swine Research Station, University of Guelph, which is a 300-sow operation that practices monthly batch farrowing. All animals used in this trial were Landrace x Yorkshire gilts. At 6 months of age, puberty was induced in all gilts by a single IM injection (5 mL) of 400 IU of eCG and 200 IU of hCG (PG600^®^, Merck Animal Health, Kirkland, QC, Canada). In addition, gilts were then provided with 20 min of boar exposure per day. When gilts were observed in estrus for the first time, they were moved to stalls and given a daily 6.8 mL oral dose of altrenogest (2.2 mg altrenogest/mL, Regu-Mate^®^, Merck Animal Health, Kirkland, QC, Canada) for a minimum of 14 d to suspend cycling. At this stage, gilts were randomly assigned to one of three treatment groups using systematic random sampling.

Group 1 (LUT, *n* = 62) was given an IM injection with 600 IU of eCG (Pregnecol^®^, Vetoquinol, Lavaltrie, QC, Canada) 24 h after the last dose of altrenogest plus 5 mg of pLH (Lutropin^®^, Vetoquinol, Lavaltrie, QC, Canada) 80 h after eCG. Dosages of Pregnecol^®^ and Lutropin^®^ were based on previous studies [4,10,11]. Group 2 (TRI, *n* = 61) received a 2 mL intravaginal dose of triptorelin acetate (OvuGel^®^, 100 μg triptorelin per mL, Elanco, Guelph, ON, Canada) 6 d after the last dose of altrenogest. Group 3 (CON, *n* = 57) served as the control group. A single FTAI of approximately 3 billion sperm was performed 36 h after the pLH injection in LUT, 24 h after the triptorelin acetate treatment in TRI, and at first sign of estrus in the CON group. CON animals received a second insemination 24 h later if the gilt was still in standing heat. Of the 60 gilts that were recruited into this group, three were not bred due to the lack of signs of standing heat. For all treatment groups, inseminations were done with a boar present in order to stimulate estrus behavior. Time of ovulation was monitored by transabdominal ultrasonography (Honda HS-1600 scanner, Honda Electronics, Tokyo, Japan) to confirm time of ovulation in gilts from each treatment group (LUT *n* = 10, TRI *n* = 8, CON *n* = 8). This was done in 12 h intervals starting on Day 5 after altrenogest was withdrawn and at 4 to 6 h intervals starting on Day 6 until ovulation was complete [10,12].

Data recorded included: gilt identification, treatment group, batch number, date of treatments, breeding date(s), farrowing date, piglet birth weights, total number of piglets born, litter characteristics, weaning date, and piglet weight at weaning. Cross fostering was avoided and weaning was done at the same time for the entire batch at approximately three weeks after the expected farrowing date for the majority of sows, which is a common weaning age in North America and a standard procedure at the Arkell facility.

### 2.2. Data Analysis

Data were analyzed by Stata (Stata/SE 14.2 for Mac; StataCorp, College Station, TX, USA). Significance was determined at *p* < 0.05. Descriptive statistics, such as means, standard deviations, and proportions, were calculated for the outcomes of interest listed above. The number of breeding days in CON gilts and the time of ovulation in all groups were reviewed. The time (in days) from when the first gilt in a treatment group farrowed until the last gilt in a treatment group farrowed was determined. The range of weaning weights among piglets, as well as the proportion of pigs with a weaning weight of less than 4.5 kg, in each group was also determined.

Differences in the likelihood of farrowing, litter size, and piglet weaning weights among the treatment groups were modeled separately. Pearson correlation analysis was utilized to examine for colinearity among normally distributed variables. Lowes curves were visually examined for linearity of x-variables. All plausible interactions were assessed and post-estimation diagnostics were performed.

The likelihood of farrowing (1 = yes, 0 = no) was examined using logistic regression (maximum likelihood estimation) with batch modeled as a random effect. Univariable associations between treatment group and other potential covariates with the likelihood of farrowing were initially investigated. Significant univariable associations at a liberal *p*-value (*p* < 0.20) were considered for the model and introduced in a forward stepwise method. The likelihood ratio test was utilized to assess significance of categorical variables in the model. Transformations of covariates were considered where appropriate (i.e., examination of the addition of a quadratic term). Litter size (count) representing the total born was analyzed using a Poisson regression model and was built in a similar manner as described above. The association of piglet weaning weight (continuous variable) with treatment group was assessed using a linear regression model with pen and batch modeled as random effects (pen nested within batch) and was built in forward stepwise fashion similar to described above. Post-estimation diagnostics were performed independently for each model type and supported optimal model fit.

## 3. Results

The farrowing rate for LUT, TRI, and CON was 75.8%, 80.3%, and 89.5%, respectively. The range in farrowing dates among treatment groups for each monthly farrowing batch showed that LUT gilts were typically the first to farrow and also farrowed within the fewest number of subsequent days compared to TRI and CON gilts. The range of breeding dates for CON gilts was an additional day and a half longer compared to the FTAI groups.

The multi-level mixed effects linear regression model (Table 1) indicated that the weaning weights of piglets in LUT and TRI were positively influenced by the use of FTAI protocols. There was no significant difference between the weaning weights of LUT and TRI piglets (*p* > 0.05). After controlling for birth weight, the piglets in LUT were 80 g heavier at weaning than the piglets in CON (*p* < 0.001). The piglets in TRI were also heavier than the piglets in CON by 64 g (*p* < 0.05). The proportion of small pigs at weaning (<4.5 kg) were 14%, 17%, and 26% for LUT, TRI, and CON, respectively (*p* < 0.05 for CON compared to others). Although CON had a farrowing rate that was up to 14% higher, and its gilts produced one to two additional piglets per gilt compared to the treatment groups, the regression models indicated that the likelihood of farrowing and litter size were not associated with the treatments (*p* > 0.05).

Detailed ultrasound results of time of ovulation in gilts revealed that five of eight CON gilts had not ovulated until 26 to 33 h after the first breeding, whereas all FTAI gilts (LUT *n* = 10, TRI *n* = 8) ovulated between 4 to 26 h post-breeding. The farm protocol was to wean the entire batch on the same day and so weaning age varied, with some litters being younger than 21 d of age.

## 4. Discussion

One of the most difficult tasks on a breeding farm is to have a consistent supply of service eligible gilts to insert into each breeding batch to replace the sows that need to be culled. Weaned sows typically return to heat as a synchronized group within 1 or 2 d of each other, but estrus in gilts is much more difficult to control. A program of boar exposure and a single injection of eCG plus hCG (PG600^®^) was used to induce puberty and estrous cycles were synchronized in gilts by a daily oral dosing of altrenogest. The cessation of the altrenogest regimen was used to provide gilts that were expected to come into heat within 4 to 9 d. In this trial, two different techniques were used to manipulate the breeding of gilts in an attempt to match the schedule of breeding weaned sows. By inducing ovulation and using FTAI it was reasoned that if breedings all occurred within 24 h, then farrowings should also occur within a few days of one another. The advantages of all sows farrowing close together includes improved labor efficiency in the farrowing room due to easier cross-fostering, better supervision of farrowing, efficient processing of litters, and less variation in piglet ages and weights at weaning. In this study, the number of subsequent farrowing days across treatment groups indicated that LUT and TRI gilts had narrower ranges of farrowing dates compared to CON gilts. Furthermore, LUT gilts farrowed in a shorter interval than TRI gilts.

When females farrow close together, the lactation length will be similar among them as well as the age and size of their piglets at weaning. Both FTAI groups had older and heavier pigs at weaning compared to CON pigs, which is consistent with the number of breeding dates required for each treatment group. Batch farrowing is more acceptable to most producers if they can control and maintain uniformity in lactation length and weaning weights within the batch [5]. The impact of consistent age and size at weaning results in less variation in the weights of pigs through the subsequent growing phases and reduces the need to provide special care for the under-weight pigs at weaning [5]. Using the weaning weight range from each treatment category, we can conclude that LUT gilts achieved a narrower range of weights among piglets compared to the conventional method of breeding (CON). In some herds, pigs under 4.5 kg will not be weaned or nurseries will not accept such small pigs. There is often an economic incentive not to wean lightweight pigs (<4.5 kg). In the present study, CON gilts had the highest proportion of lightweight pigs at weaning compared to LUT and TRI (*p* < 0.05). This is a result of the greater variation in breeding and farrowing dates. Piglets from gilts bred by FTAI (particularly eCG–pLH) were more uniform in size and the average weaning weight was heavier than piglets from gilts bred by traditional methods. This has economic significance because an increased weight at weaning better prepares pigs for successful performance in subsequent stages of production [7]. The fact that pigs with heavier weaning weights grow faster post-weaning has been well established [7]. It has been reported that, compared to pigs weighing 4.1 to 5.5 kg, pigs that weighed 6.8 to 8.2 kg at weaning reached 105 kg about 10 d earlier [8].

A secondary objective of this study was to investigate the effects of hormonal induction of ovulation by eCG–pLH or triptorelin acetate on reproductive performance. CON gilts had a numerically higher farrowing rate compared to LUT and TRI. This is consistent with a previous study in which the farrowing rate of gilts that received pLH and a single FTAI was decreased compared to control gilts that received multiple inseminations [9]. However, it was suggested that this could be a result of improper timing of pLH administration since fertility is affected by follicles that are not sufficiently mature before the LH surge occurs [9]. In addition, a review by De Rensis and Kirkwood [4] also stated that poor fertility was the result of a similar protocol involving eCG plus hCG followed by pLH 80 h later in prepubertal gilts.

Of note, based on the protocols used in the present study, all gilts that were assigned to LUT and TRI were bred, but in CON if gilts failed to show signs of estrus, then they were not bred. Therefore, if subfertile animals were assigned to LUT or TRI they were factored into the farrowing rate for those groups but if they were assigned to CON they were likely removed by not showing heat. Therefore, it is possible that one should compare the number of successful farrowings to all gilts assigned to a treatment group instead of using farrowing rate as the reproductive parameter to measure success. However, in this study, only three gilts in CON were not bred and, therefore, this may not have made a difference in the overall conclusions in this case.

The effect that eCG–pLH and triptorelin acetate had on litter size was also of interest in the present study. The regression models indicated that neither treatment had an impact on litter size. However, it is important to mention that numerically, CON gilts had a litter size of one to two additional piglets per litter compared to LUT and TRI. This suggests that a future study using a larger sample size is warranted to determine if FTAI protocols do reduce litter size and to determine the mechanisms involved.

Ultrasound assessment revealed that LUT and TRI gilts ovulated within a timeframe that would allow for a successful single, fixed-time insemination. The majority of CON gilts did not ovulate until 26 to 33 h after the first breeding as expected. This demonstrates that the conventional approach of using a second insemination 24 h after the first breeding is necessary and appropriate for ensuring a good conception rate. The ovulation results of the present study are similar to those of other studies. In a study that used a GnRH agonist in eCG estrus-synchronized gilts, the time of ovulation, as determined by repeated endoscopic observation, began between 38 and 42 h after the GnRH agonist treatment and was completed in 50% of the gilts by 42 h (10). Using transabdominal real time ultrasonography, another study found that of gilts that received pLH at the onset of estrus, 70% ovulated within 36 h compared to 52% of gilts in the control group (9).

In conclusion, the FTAI protocols used in this study resulted in a narrower range of farrowing dates and the weaning weights of piglets from these litters were statistically heavier than piglets from gilts bred by conventional methods. This has the potential for economic reward, but caution is required as the present study also provided evidence that reproduction parameters such as litter size and farrowing rate tended to be lower than controls. The FTAI protocols used in this study were based on procedures used in previous research using multiparous sows. Future research needs to be performed to determine if timing and dosage might be modified to improve reproductive performance in gilts. In addition, there may also be farm-to-farm differences and this should be evaluated before recommendations regarding FTAI protocols for gilts are made.

## Figures and Tables

**Table 1:** The final model ˚ illustrating the effect of fixed-time artificial insemination (FTAI) (LUT and TRI *) versus conventional breeding on the piglet weaning weight (g) for gilts.

Variable		Weaning Weight (g)	SE	95% CI	*p*-Value
Treatment *	CON (referent)				
	LUT	79.7	22	37, 123	<0.001
	TRI	63.7	22	19, 107	0.004
Birth weight		364.5	15	336, 393	<0.001

˚ Multi-level mixed effects linear regression model, pen and batch modeled as random effects. * LUT: eCG + pLH and FTAI 36 h later. TRI: triptorelin acetate and FTAI 24 h later. CON: control. SE: standard error. CI: confidence interval.
