# Erratum: Rodrigues, L.; Amezcua, R.; Cassar, G.; O’Sullivan, T.; Friendship, R. Comparison of Single, Fixed-Time Artificial Insemination in Gilts Using Two Different Protocols to Synchronize Ovulation. *Animals* 2020, *10*, 306

**DOI:** 10.3390/ani10040641

**Published:** 2020-04-08

**Authors:** Lima Rodrigues, Rocio Amezcua, Glen Cassar, Terri L O’Sullivan, Robert Friendship

**Affiliations:** Department of Population Medicine, University of Guelph, Guelph, ON N1G2W1, Canada; lrodrigu@uoguelph.ca (L.R.); mamezcua@uoguelph.ca (R.A.); tosulliv@uoguelph.ca (T.L.O.); rfriends@ovc.uoguelph.ca (R.F.)

The authors wish to make the following corrections to their paper [1]:

The heading “Conclusions” should be inserted before the final paragraph of the Discussion.

The following citations in the ‘Discussion’ should be changed:

[5] replaced by [7].

[7] replaced by [13].

[8] replaced by [14].

[9] replaced by [5].

[10] replaced by [15].

In the Discussion, in the paragraph which begins with ‘Ultrasound assessment’, a new citation, [10], should be inserted after the following sentence: The ovulation results of the present study are similar to those of other studies.
